# Evaluating the effectiveness of the Family Connections program for caregivers of youth with mental health challenges, part II: A qualitative analysis

**DOI:** 10.1111/hex.13220

**Published:** 2021-02-24

**Authors:** Natasha Y. Sheikhan, Karen Wang, Tali Boritz, Lisa D. Hawke, Shelley McMain, Joanna Henderson

**Affiliations:** ^1^ Centre for Addiction and Mental Health Toronto Ontario Canada; ^2^ Dalla Lana School of Public Health University of Toronto Toronto Ontario Canada; ^3^ Department of Psychiatry University of Toronto Toronto Ontario Canada; ^4^ Department of Psychiatry Sunnybrook Health Sciences Centre Toronto Canada

**Keywords:** adolescent, caregivers, family relations, mental health, peer group, qualitative research

## Abstract

**Background:**

The Family Connections™ (FC) program is a 12‐week support and skill‐training program for caregivers of youth with mental health challenges. The intervention was originally developed with a focus on borderline personality disorder (BPD). It is important to understand the experiences of caregivers in such interventions, as well as its applicability beyond BPD, for the purposes of evaluation and ongoing program improvement.

**Objective:**

To explore and analyse the experiences of caregivers of youth with diverse mental health challenges and who participated in FC.

**Design:**

Semi‐structured interviews with thirteen FC‐participating caregivers of youth with mental health challenges.

**Results:**

Thematic analysis uncovered three major themes regarding caregivers' experience with FC: (a) FC increased the caregivers' ability to manage their youth's mental health challenges; (b) participating in FC impacted their intra‐ and interpersonal spheres; and (c) improvements to the program were proposed. Following participation in FC, caregivers felt they learned a new approach to understanding themselves, their youth and mental health, and were better able to manage their youth's mental health challenges.

**Discussion and conclusion:**

FC is a promising intervention for caregivers of youth with mental health challenges, beyond the traditional BPD focus. The intervention has the potential to provide broad‐based benefits for caregivers and should be considered for implementation and scale‐up across youth‐ and caregiver‐serving organizations. Potential areas of intervention flexibility and improvement are discussed.

**Patient/public contribution:**

Caregivers were involved in the program development and facilitation of FC. A person with lived experience was involved with the analysis.

## 1 INTRODUCTION

Caregivers of individuals with mental health challenges, ranging from general distress to mental disorders, experience higher rates of mental health challenges compared to the general population.^1^ Many feel confusion, fear, distress, and a sense of grief regarding their loved one's mental health challenges. Furthermore, caregivers face significant systemic challenges, often lacking the necessary mental healthcare resources; for example, they may face barriers accessing care and navigating changes to service delivery, while also experiencing stigma.^2^, ^3^, ^4^, ^5^ Caring for youth with mental health challenges can also present unique challenges, especially as 18%‐22% of youth ages 12‐17 years old meet the criteria for a mental disorder.^6^ Caregivers often undertake unfamiliar roles and responsibilities with regard to supporting their youth, which can generate distress in their own lives. As a result, caregivers of youth with mental health challenges report high rates of caregiver burden, feelings of helplessness and ineffectiveness, and distress.^1^, ^7^ Caregivers of youth with mental health challenges can also experience financial strain and heightened stress due to their youth's mental health challenges.^8^

In order to support caregivers of youth with mental health challenges, multiple intervention models have been developed. Notably, peer‐based programs providing education and support for caregivers have demonstrated positive impacts.^9^, ^10^, ^11^ Peer‐based programs include a range of interventions entailing interactions between people who share lived experience on a specific issue.^12^ In peer‐based mental health services, such as peer education and support programs, participants show improved self‐reported empowerment and hope along with increased self‐esteem.^13^, ^14^ Moreover, peer‐based programs can be cost‐effective.^15^, ^16^ Involving peers in health comes with benefits that may not be as prominent in traditional clinical‐led models, such as fostering trust and rapport with participants.^11^ As a variation of peer‐based programs, team‐led programs are also available. Team‐led programs are co‐delivered by peers and mental health professionals together, a model that has also demonstrated benefits.^17^

Family Connections (FC) is a manualized peer‐based education, skills training, and support program originally created for family members of individuals with borderline personality disorder (BPD). This support model is typically implemented in groups within a community setting and focuses on providing information and skills training to improve family functioning, coping skills, and provide social support.^18^ ^40^The FC model is based on skills and strategies drawn from Dialectical Behavior Therapy (DBT)^19^ and includes psychoeducational components, skill‐building, and support. It is informed by DBT's biosocial theory, which highlights the transaction between a person's biology (e.g. predisposition to emotional vulnerability) and their developmental environment. Accordingly, the FC group balances support for participants' well‐being by teaching them skills to understand their family members and modify their behaviours and responses.

Previous studies evaluating the FC program illustrate benefits ranging from reducing grief levels to improving participants' sense of their knowledge and coping skills and improving family function.^18^, ^20^, ^21^, ^22^ The FC program's impact on participant well‐being has mainly been addressed through quantitative studies, focusing predominantly on BPD.^18^, ^20^, ^21^, ^23^ An initial study by Hoffman et al^23^ evaluated the FC program among family members of individuals with BPD, showing increased mastery skills and reduced levels of grief among the participants after the program. Hoffman et al^18^ replicated the original findings in another study, showing significant improvement in well‐being and reducing depression among participants. Rajalin et al^22^ conducted a pilot study evaluating FC among family members of individuals who had attempted suicide, finding a significant reduction of the sense of burden and improved well‐being. Another study looked at the effectiveness of FC vs. an optimized treatment as usual for family members of individuals with BPD.^20^ Consistent with the aforementioned studies, Flynn et al^20^ found significant reductions in the sense of caregiver burden, grief, and depression, along with an increase in mastery among the FC group. More recently, Liljedahl et al^21^ evaluated the standard FC training (FC‐S) compared to an intensified weekend training (FC‐R). Both groups showed significantly improved functioning, overall family function, and perceived resources in caring for significant others with BPD.^21^

Although these studies demonstrate the positive impacts of the FC program in relation to BPD, there is a dearth of information on participants' qualitative experiences of the program, including caregivers supporting youth with mental health challenges outside of the BPD sphere; thus, it remains unclear which elements of the program contribute to these positive outcomes, and whether these findings extend beyond BPD Accordingly, the present study aimed to explore caregivers' experience of an FC program designed for caregivers of youth with diverse mental health challenges, providing a qualitative lens on FC impacts for caregivers outside of the domain of BPD.

## 2 METHODS

### 2.1 Study setting

This study was part of the Research and Action for Teens (RAFT) study, a multi‐pronged, multi‐cente program of research that included an effectiveness evaluation of the FC program adapted for caregivers of youth with mental health challenges. The RAFT study was conducted across three cities in Ontario, Canada (Toronto, Ottawa, and Thunder Bay).^18^, ^24^ Participants in the RAFT Family study were recruited through flyers, notice boards, and email servers across all three communities to participate in the FC program and associated research project. Inclusion criteria were as follows: (a) 18 years of age or older, (b) literacy in English, (c) family member or caregiver of an adolescent (ages 14‐18) scoring in the clinical range on at least one subscale of the Child Behavior Checklist (CBCL), as reported by the participating family member; caregivers reported a variety of youth disorders represented in the sample, and (d) did not have an adolescent child participating in the other arm of the RAFT study providing a youth intervention. Individuals interested in participating received a full description of the study and were screened by a research assistant to determine eligibility. Written informed consent was obtained from participants prior to enrolment. A quantitative evaluation of the RAFT Family study is provided in a companion manuscript.^25^

As part of the RAFT Family study, the current qualitative sub‐study took place at Toronto and Ottawa sites. The Thunder Bay site was not included in the qualitative study due to logistical issues associated with conducting in‐person interviews in this location. In this study, interviews were conducted with participants who took part in the FC program post‐treatment. All caregivers actively taking part in the RAFT Family study in Toronto and Ottawa between April 2013 and May 2014 were invited to take partin the qualitative study. All of the interviews were carried out by KW, a psychiatry resident at the time.

### 2.2 Sample

The sample of the current study consisted of 13 caregivers who participated in the RAFT Family study (Toronto and Ottawa locations) and consented to be interviewed. The invited participants were distributed among the two treatment sites (Toronto = 10; Ottawa = 3). Of the 13 participants, seven were female, and six were male. The participants ranged in age from 40 to 55. See Table 1 for additional demographic information.

**TABLE 1:** Demographic characteristics

	Toronto—10	Ottawa—3
Gender		
Male	5	1
Female	5	2
Age		
<40	0	0
40‐45	4	0
46‐50	3	2
51‐55	3	1
Caregiver ethnicity		
White	10	3
Other	0	0
Youth ethnicity		
White	9	3
Other	1	0
Marital status		
Married	7	3
Separated	2	0
Divorced	1	0
Highest education level		
Elementary	2	0
High School	7	3
College	1	0
Employment status		
Full time	9	2
Part time	0	1
Not working	1	0
Household income		
$10 000‐$29 999	1	0
$30 000‐$59 999	1	0
More than $60 000	8	3
Number of family members in household		
1‐2	1	0
3‐4	8	0
5+	1	3

### 2.3 Intervention

The FC program was based on Fruzzetti & Hoffman's^26^ FC program and adapted for family members of youth with mental health challenges.^26^ FC is a 12‐week group‐based intervention traditionally led by trained family members with personal experience supporting a loved one with mental health challenges, in community settings. However, in this study, groups in Ottawa were peer‐led in a community‐based peer‐run organization, while the groups in Toronto were team‐led (by a caregiver and a service provider) in a tertiary care center, as an adaptation to meet local organizational requirements to permit implementation. Each group session ran for approximately 90 minutes. All family members received a manual describing the skills covered. A 2‐day training in the FC model was completed by all facilitators. The training was led by one of the treatment developers, and consultation was provided by DBT therapists with intensive training in the FC model. For more information about the FC program, refer to *National Education Alliance for Borderline Personality Disorder (NEA‐BPD) Family Connections* (https://www.borderlinepersonalitydisorder.org/family‐connections/).

### 2.4 Data collection

Caregivers took part in semi‐structured interviews at the end of the FC program (i.e., after 12 weeks). Semi‐structured interviews were conducted from April 2013 to May 2014. Interviews ranged from 50 to 105 minutes in duration. The semi‐structured interview guide consisted of open‐ended questions related to the caregivers' experience with their youth's mental health challenges, including questions about their caregiving experiences, the impact of the FC training, and their experience attending the FC group. The interviews were audio‐recorded, transcribed verbatim, de‐identified, and entered into qualitative analysis software (NVivo 12^27^) for analysis.

### 2.5 Data analysis

The primary a priori defined topic that we examined was the impact of the FC program on caregivers. Data were analyzed using thematic analysis through an interpretive process.^28^ All transcripts were analyzed and coded by one member of the research team with relevant lived experience, who was a graduate student at the time. The codes were refined through collaborative and in‐depth discussions, and a consensus of codes was interpreted during the analytical process. Inter‐rater reliability was established on 20% of the transcripts with a second coder. The coding process was conducted collaboratively through in‐depth discussions among team members with backgrounds in psychology to generate themes and subthemes that guided the interpretation of the data. The analysis closely followed four phases of theme development: (a) initial identification of possible themes; (b) the articulation and construction of themes; (c) relating themes back to existing knowledge; (d) developing a logic or storyline from themes related to our research question.^28^ Key quotes were selected from the data and presented to further illustrate findings.

The consolidated criteria for reporting qualitative research (COREQ) guidelines were followed to ensure a comprehensive reporting of our study.^29^ Research Ethics Board approval was obtained from the Centre for Addiction and Mental Health (CAMH) and the Royal Ottawa Health Care Group Research Ethics Board. Family members within the RAFT study were engaged as service providers.

## 3 RESULTS

Our analysis identified three major themes regarding caregivers' experience with the FC program: (a) the intervention increased caregiver's ability to manage their youth's mental health challenges; (b) participating in the program enhanced caregiver's inter‐ and intra‐personal spheres; (c) caregivers' experience with the program led to proposed improvements to the program. Emerging from the analysis were several subthemes that helped capture the complexity of each key theme as it relates to the FC program.

### 3.1 The intervention increased caregiver's ability to manage their youth's mental health challenges

Caregivers experienced a new understanding of their youth's mental health challenges, increased effectiveness in response to their youth's mental health challenges, and a positive shift in their caregiving experience. Through these experiences, caregivers increased their ability to manage their youth's mental health challenges.

#### 3.1.1 New understanding of their youth's mental health challenges

In participating in the FC program, caregivers reported a shift in the conceptualization of their youth's mental health challenges. Prior to the FC program, caregivers endorsed a range of negative emotions related to their youth's mental health challenges, including frustration, depression, guilt and resignation (e.g., the urge to give up). One respondent, reflecting on their emotions prior to going through the FC program, said:

We were almost like, ready to wash our hands and say, “I can't do anything.” And I still can't believe that the thought crossed our minds at one point. (Participant 1)

Almost all caregivers reported an expanded understanding of their youth's mental health challenges. For example, one caregiver shared that prior to the FC program, they interpreted their child's behaviour as something she was doing to ‘torture’ their family. Following the program, they saw her behaviour as something ‘not necessarily in her control…she's just wired that way’ (Participant 1). Others noted seeing signs of mental health challenges but not fully making sense of them before the FC program. Through the FC program, these caregivers reported a new understanding of the issues underlying their youth's behaviour, which shifted some of the emotions that had previously been activated.

Caregivers reported increased feelings of acceptance during and following the FC program. Some caregivers felt more accepting of their youth's mental health challenges, their roles as caregivers and their youth's future (e.g., the likelihood that mental health challenges would persist). Other caregivers described feeling hopeful about their youth's future and prognosis, as well as their ability to handle their caregiver role. Despite sharing many positive shifts in emotions, a few caregivers continued to express ongoing frustration in response to their youth's mental health challenges, particularly related to behaviours they found particularly problematic.

#### 3.1.2 Increased effectiveness in response to youth's mental health challenges

Caregivers reported feeling more effective at responding to mental health challenges when caring for their youth as a result of the FC program. Many reported that prior to the FC program, their typical reactions included feeling confused about how to manage new problems or crisis situations arising from their youths' mental health challenges. This led to frustrating and defensive encounters with their youth and with mental health services. For example, some caregivers resorted to strong measures, such as contacting law enforcement as a response to their youth's mental health challenges, which they later regretted because it often served to compound rather than diffuse a specific issue or situation. The caregivers who reported involving law enforcement stated this was predominantly rooted in uncertainty about how to respond to a mental health crisis:

We knew it was wrong. It didn't work. Not everything works with every child, but we didn't know what to do, because we didn't understand enough about – even though we were reading up on it. (Participant 5)

As a result of participating in the FC program, caregivers felt more effective and confident in their ability to manage their responses should mental health challenges arise in the future. For example, caregivers stated that they now know how to prevent situations from escalating and how to apply strategies in the moment to decrease the likelihood of a negative outcome. Participants also discussed feeling less dysregulated and calmer in crisis situations after participating in the FC program. Reflecting on their shift in reactions, one caregiver shared:

It's not ‘Okay, what do I need to do now?’ It's more “Okay, […] she's upset […], she wants to stay out all night. It must be hard that your friends are allowed to stay out ‘till 6 o'clock in the morning and you're having a fun time and you feel like you're going to be left out. Or you feel like […] you're immature because you have to go home.” Like ‘I'm sure that's really difficult for you.’ And those are things that I wouldn't have really thought about at other times, […] prior to the training. (Participant 11)

Caregivers in the FC program felt the intervention provided them with skills that had been available to them in their previous encounters with the healthcare system. An important area of skill development was being able to better react and respond to challenging, often new and unsettling situations with their youth. Although some found practising the skills challenging, caregivers felt they had a clearer understanding of the challenges their youth were experiencing, which helped increase their effectiveness in providing support to their youth.

#### 3.1.3 Positive shifts in their caregiving experience

Participants also reported a positive shift in their caregiving experience. Many caregivers reflected on prior negative experiences with their youth, which included attempts to enforce strict or controlling disciplinary strategies and frequent arguments. Less often, caregivers reported avoidance behaviour, such as avoiding their youth during conflict. Caregivers also reported intimidation and problematic contingencies, such as unhealthy negotiation, that is, bribing their youth to behave better.

As a result of the FC program, participants expressed that they had learned to better communicate with youth, which included both listening more closely and effectively communicating their feelings to their youth. As caregivers worked through the FC program, many identified specific skills (e.g., validation), which enhanced communication with their youth. Some even expressed applying these skills in other areas of their lives (i.e., outside of the caregiving context). One caregiver shared how learning to validate feelings shifted his approach to caregiving:

It's just validation defusing when he's really, really angry, […] taking a little bit of time and recognizing or validating why he wanted the car and why it was important for him, and then sort of talking about my concerns. The big ‘ah ha’ in there was, that was the first time that I could actually get him to sit down and listen to what my issues were, because he would usually go into a rage and then we couldn't have a conversation. (Participant 12)

Most of the caregivers discussed applying the skills learned from the FC program to their daily caregiving routine. Several caregivers described themselves as more lenient and patient in their caregiving style. They also endorsed a decrease in their withdrawal behaviours, while simultaneously giving their youth more space.

### 3.2 Participating in the program enhanced caregiver's inter‐ and intra‐personal spheres

During and after their participation in the FC program, caregivers experienced a better relationship with their youth, a sense of belonging through their relationships with other FC participants, and an increase in self‐awareness and personal control. Overall, engaging in the FC program enhanced caregivers' inter‐ and intra‐personal spheres.

#### 3.2.1 Better relationship with youth

Caregivers experienced a shift in their interpersonal sphere, with almost all participants reporting a better relationship with their youth following the FC program. Many discussed having a strained relationship with their youth prior to the FC program, including high levels of conflict and a sense of ‘distance’ in the relationship. Reflecting on their experiences after the FC program, caregivers reported feeling more willing to provide frequent support to youth. Some noted that this shift was accompanied by an acknowledgement from their youth that they felt more supported and understood by their caregivers.

Participants credited their enhanced ability to communicate with their youth as positively influencing their relationship. For example, they observed a reciprocal shift in their youth's behaviour, with their youth opening up and communicating more. Many of the caregivers described feeling a more loving relationship with their youth after the FC program. Some even reported greater instances of physical demonstrations of affection:

It's totally different. She hugs me good‐bye. She'll give me a hug, like ‘Thanks for the movie’. […] That's definitely partly a product of me digging in and applying some of these concepts. Yeah, that wouldn't have happened half a year ago — for sure not. (Participant 6)

#### 3.2.2 Sense of belonging

Most caregivers expressed they felt less alone as a result of their participation in the FC program. Some appreciated hearing their experiences reflected by other group members' experiences during the group discussions. Caregivers repeatedly described the positive impact of sharing similar emotions and experiences with participants struggling with similar issues to them. Many reflected that prior to their participation in the FC program, they had not felt their experiences were relatable, contributing to their sense of isolation and aloneness. Some caregivers reported the connection they felt to their group members extended beyond the group itself, with some participants continuing to get together after the program ended. A few caregivers shared that this sense of belonging had begun with their first FC session:

The first class was great, because all of a sudden, we weren't alone […]. (Participant 8)

In addition to feeling a sense of belonging, caregivers reported feeling empowered by their shared experiences in the group. One caregiver described:

We looked forward every single Saturday to getting there, to learning more. Every time we would go, we left feeling like the weight of the world was lifted from your shoulders, because there's other parents going through what you're going through and we felt empowered because we were learning. […] The parents understand, and you feel like you're helping your child. And it's made a huge difference in our home. (Participant 5)

#### 3.2.3 Increased self‐awareness and personal control

Many caregivers felt they were coping with their distress better as a result of participating in the FC program. For example, participants described using self‐care strategies they learned from the FC program and that they continued to develop after the program was complete, including exercise, mindfulness, going for walks and taking time to self‐reflect. One caregiver described their self‐care strategies post‐FC:

I just go for that walk. […] I'll sit on a bench and watch people. So, that's the way I kind of reflect and sort out things and just be by myself. […] I find that's really, really been therapeutic for me. (Participant 3)

Caregivers further reported a significant shift in prioritizing their self‐care following the FC program, including spending more time socializing with friends. One caregiver reported that their improved self‐care was associated with sleeping better and feeling calmer. Other caregivers disclosed feeling less guilty both about past experiences with their youth and about setting time aside for themselves in the present.

Many of the caregivers reported that the tools they learned in the FC program helped them to feel more in control, with increased insight into their former caregiving behaviours. One caregiver shared:

I feel empowered. I feel more in control—not of the situation, but of the way that I can feel about it. […] I was completely at a loss and […] now I feel like I can do this. (Participant 11)

Some caregivers expressed increased self‐understanding through the process of attending the FC program:

That is the most enlightening and best part of the sessions. Listening to the stories and the situations that they were in. […] It was the stories from the parents that were the most enriching — and what worked and what didn't work. And my telling my story, because I find the more I go through things in my mind and then verbalizing things, the more validation I get from it and the more that I learn about myself and about the situation. (Participant 3)

Caregivers developed a greater understanding of their own mental health challenges and general state of mind. Overall, caregivers described the program as introducing them to a new spectrum of knowledge and a way to understand themselves and others.

### 3.3 Caregivers' experience with the program led them to propose improvements

Caregivers' experience with the FC program shaped their recommendations around improvements to materials and facilitation, program structure and program components.

#### 3.3.1 Materials and facilitation

Caregivers expressed appreciation around the psychoeducational materials and style of the facilitators. However, participants also suggested improvements to the group materials. For example, one caregiver expressed wanting to see more examples in the information sheets and handouts paired with the 12‐week curriculum. Another participant suggested having the materials emailed to them prior to the sessions so they could review them in advance.

Caregivers discussed a positive experience with the program facilitation. Many caregivers in the Toronto group highlighted the value of the team‐led approach. Other caregivers in the Toronto group expressed the benefit of gaining skills from the professional facilitator, with some hesitation about the possibility of a peer‐led facilitation model, which they did not experience. Conversely, in the Ottawa group, which was peer‐led, caregivers expressed appreciation around the peer‐led approach:

I think these people were experts. Plus, they have… they've seen a lot. […] Almost everything that we've seen, they've seen. And they can relate. But they kept things moving. And they don't let you dwell too much on that kind of stuff, right? When they went through all the different, uh, I don't think you could have done much different or much better. (Participant 9)

#### 3.3.2 Program structure and components

A common suggestion about improving the program was to prolong the length of the sessions and program itself. Some caregivers suggested ideally extending the program past 12 weeks. One respondent shared:

I think in the ideal world, you would probably have about 16 of these sessions and then just follow ups, so maybe meetings once a week, or sorry, once a month or something, because you have to keep practicing it. Because the parents who are in these groups, they're under a lot of stress. Yes, we're there for 2 hours, but our learning capacity is not what it is when we have healthy children. (Participant 13)

A few caregivers proposed additional content, such as additional stand‐alone sessions on mindfulness and self‐care. One participant suggested that time might be reserved at the end of each session to do the assigned homework rather than requiring participants to take it home; they also suggested that even having time after class to simply reflect would be useful. One caregiver shared how they would have enjoyed an FC blog or conference to further discuss the material.

A few caregivers found aspects of the sessions to be overwhelming. For instance, some participants found that listening to other parents' experiences was overwhelming or distressing and brought up emotions they experienced as difficult or painful. They felt that extra supports could be offered to offset this potentially overwhelming experience.

Overall, the majority of caregivers enjoyed the program and would recommend it to others. For instance, one participant shared:

I would highly recommend it, just — even if you aren’t able to learn the skills, just be in the room with other people who understand your experience. (Participant 2)

Another suggested that the FC program be made more widely available and accessible.

## 4 DISCUSSION

This qualitative study sheds light on the experience of caregivers of youth with mental health challenges who participated in a 12‐week DBT‐based skills training and support intervention for family members. In extending prior knowledge on support services for caregivers, this study shows the positive impacts of the FC program for caregivers of youth experiencing a range of mental health challenges. The analysis uncovered three major themes regarding caregivers' experience with the FC program: (a) FC increased the caregivers' ability to manage their youth's mental health challenges, (b) participating in FC impacted their intra‐ and interpersonal spheres, and (c) improvements to the program were proposed. Participants experienced improved reactions to their youth's mental health challenges, while re‐conceptualizing mental health challenges in general and the mental health challenges of their youth in particular. They also experienced a positive shift in caregiving as a result of the FC program. Participants further shaped recommendations for program improvement.

Family members of youth with mental health challenges have been previously found to experience distress, guilt, helplessness, blame and shame.^30^, ^31^, ^32^, ^33^, ^34^, ^35^ Previous research has demonstrated that caregivers of youth experience improvements in both well‐being and functioning as a result of the FC program, primarily focusing on BPD.^18^, ^20^, ^22^ These findings are reflected and extended upon in the current study, as caregivers of youth with mental health challenges described experiencing various forms of emotional distress and burden before the FC program and reported substantial improvements in these areas in association with the program. In terms of functioning, Liljedahl et al^21^ found improvements in overall family function after the FC program among caregivers. Similarly in an evaluation of a peer‐led program for caregivers of children with mental health challenges, Brister et al^36^ found a reduction in inflammatory conflict (e.g. escalating conflict). The current study confirmed those findings, as caregivers reported a reduction in inflammatory caregiving responses, as well as improvements in functioning in relation to interacting with their youth and managing their youth's mental health challenges, as well as their own self‐care.

The FC program includes substantial psychoeducational and skill‐building components. Psychoeducation for families of persons with mental health challenges has been shown to have a range of perceived benefits, such as improved outcomes of care and enhanced communication.^37^, ^38^ Participating in the FC program, which includes psychoeducational and skill‐building components, provided caregivers with the information and skills they were seeking but were not otherwise available through conventional health care channels. The FC program helped them acquire knowledge that led to a better understanding of their experiences with mental health challenges, resulting in better reactions and responses to often new and unsettling situations arising from caring for a youth with mental health challenges. In terms of skill‐building, previous FC studies have found increases in mastery skills, that is, their sense of their own knowledge and coping skills, among caregivers of individuals primarily with BPD.^20^, ^21^, ^23^ Improvements in perceived skills and mastery in the current study included reframing how to think about mental health challenges, especially in a family context, as well as an increased sense of control and empowerment, and being able to let go of feelings of guilt. They gained confidence in being able to effectively relate to their youth and manage everyday life challenges.

Current family support services for caregivers of youth with mental health challenges include peer‐led, clinician‐led and team‐led program.^17^ This study employed peer‐led and team‐led models, in which the peer‐led model consisted of facilitation by caregivers with lived experience and the team‐led model consisted of co‐facilitation by a professional and a peer. Both models have been shown to be an effective approach in addressing caregiver needs; previous research suggests benefits in perceived social support and self‐efficacy among caregivers participating in team‐led programs, while peer‐led programs are associated with improvements in coping with stress, self‐care, family functioning, resilience, and communication.^9^, ^10^, ^17^, ^36^ Indeed, research shows that peer involvement in health interventions comes with benefits that may not be as prominent in traditional clinical‐led models, such as fostering trust and rapport with participants and cost‐effectiveness.^11^, ^15^, ^16^ The participants in this study pointed out that FC and peer support in general play an essential role in facilitating the development of the skills they need to re‐conceptualized and reframe their responses. Caregivers in the peer‐led model appreciated the peer‐led structure, while caregivers in the team‐led group expressed appreciation around the co‐facilitation model. The intervention was also appreciated in both a community setting (Ottawa) and a tertiary care centre (Toronto). This points to the possibility of exploring a range of peer and professional support models within the FC program, in diverse settings, as areas of flexibility to facilitate implementation in different contexts.^39^

These findings have important implications for future research and practice. FC is a manualized, implementation‐ready intervention to support caregivers of youth with mental health challenges. This study demonstrates the substantial benefits of the FC program for caregivers of youth with mental health challenges, suggesting that extensive scale‐up could provide broad‐based benefits for many caregivers of youth across the mental health spectrum, beyond the previous research focus of BPD. FC should therefore be considered for implementation and scale‐up. Those implementing and evaluating FC might consider some potential improvements highlighted by the participants, such as prolonging the length of the sessions and the program itself. The concept of belonging is a theme in our analysis and other research on group mental health interventions and peer support^39^; thus, incorporating the concept of belonging as a key component in future delivery should be considered. Moreover, although participants described FC in a positive light and notably expressed a sense of belonging in connecting with other caregivers, a few participants discussed feeling overwhelmed during the sessions. Ensuring that proper supports (e.g., clinical support workers) are in place during the sessions is critical to producing a safe environment for all participants.

Essential areas of flexibility for FC include the peer‐led vs. team‐led approaches, as well as the implementation in both community‐based and tertiary care centres, which can be chosen based on local context to support implementation. Caregiver feedback should be proactively incorporated into future iterations of the FC program. Future research should consider the barriers and facilitators to effective implementation in diverse contexts and with diverse participant groups, and also consider a more in‐depth exploration of the peer‐led vs team lead approaches. More in‐depth knowledge on the significance of programs like FC through longitudinal research design is needed to capture the changing dynamics of care, family structures and mental health challenges among youth.

### 4.1 Strengths and limitations

One of the strengths of this study is that it highlights the experiences of caregivers and their perspectives on both the value and limitations of the FC program through a qualitative approach. Involving family member consultants during the program design and an individual with lived experience during the analysis was also a strength. Moreover, as previous FC studies focus primarily on BPD, this study extends beyond that diagnostic framework to demonstrate benefits among caregivers of youth with mental health challenges in general. Lastly, a unique feature of this study is the integration of both peer‐ and team‐led approaches in community‐based and tertiary care settings. The study's positive findings across these models provide insights regarding flexible components to support future implementation efforts.

This study had several limitations. The participant sample was a small and self‐selecting group, with limited demographical differences (e.g., ethnic background). Therefore, the sample may not reflect all caregivers participating in the FC programs and limits the generalizability of these findings. Moreover, our participants were chosen from the Toronto and Ottawa locations—both large urban cities. As the FC program runs across Canada, including more remote settings, our participants may not accurately reflect caregivers' experiences in less populated and rural areas. Lastly, the peer‐led site had the smallest number of participants; this may limit the extent to which comparisons can be made between delivery models.

## 5 CONCLUSION

Our findings demonstrate the positive impact of the FC program for caregivers of youth with mental health challenges beyond the previous focus on BPD. Changes in caregiving styles, intra‐ and inter‐personal awareness, and extended models of peer‐ and team‐led approaches all play important roles. These implications highlight the FC program as a promising program that may substantially improve caregivers' lives and should therefore be considered for implementation by organizations serving youth with mental health challenges and their caregivers. Future research on family‐based studies should examine barriers and facilitators to flexible implementation and scale‐up to bring lasting effects for caregivers.

## CONFLICT OF INTEREST

The authors declare no conflicts of interest.

## AUTHOR CONTRIBUTIONS

Natasha Y. Sheikhan (joint first author) contributed to the analysis, interpretation of data, drafting the manuscript, and revising it. Karen Wang (joint first author) contributed to the conception, design, acquisition of data and revising the manuscript. Tali Boritz contributed to the conception, design, acquisition of data, analysis, interpretation of data, revising manuscript and final approval of version to be published. Lisa D. Hawke contributed to the analysis, interpretation of data, revising the manuscript and final approval of the version to be published. Shelley McMain contributed to the conception, design, acquisition of data and revising manuscript. Joanna Henderson (senior author) contributed to the conception, design, revising of the manuscript and the final approval of version to be published.

## Data Availability

The data that support the findings of this study are available from the corresponding author upon reasonable request.
